# Electroporation-based methods for *in vivo*, whole mount and primary culture analysis of zebrafish brain development

**DOI:** 10.1186/1749-8104-2-6

**Published:** 2007-03-15

**Authors:** Michael Hendricks, Suresh Jesuthasan

**Affiliations:** 1 Developmental Neurobiology Group, Temasek Life Sciences Laboratory, Research Link, National University of Singapore, 117604, Singapore; 2 Department of Biological Sciences, National University of Singapore, Singapore

## Abstract

**Background:**

Electroporation is a technique for the introduction of nucleic acids and other macromolecules into cells. In chick embryos it has been a particularly powerful technique for the spatial and temporal control of gene expression in developmental studies. Electroporation methods have also been reported for *Xenopus*, zebrafish, and mouse.

**Results:**

We present a new protocol for zebrafish brain electroporation. Using a simple set-up with fixed spaced electrodes and microinjection equipment, it is possible to electroporate 50 to 100 embryos in 1 hour with no lethality and consistently high levels of transgene expression in numerous cells. Transfected cells in the zebrafish brain are amenable to *in vivo *time lapse imaging. Explants containing transfected neurons can be cultured for *in vitro *analysis. We also present a simple enzymatic method to isolate whole brains from fixed zebrafish for immunocytochemistry.

**Conclusion:**

Building on previously described methods, we have optimized several parameters to allow for highly efficient unilateral or bilateral transgenesis of a large number of cells in the zebrafish brain. This method is simple and provides consistently high levels of transgenesis for large numbers of embryos.

## Background

Electroporation has been used successfully in chick embryos to perform gain of function (overexpression) and loss of function (dominant negative, small interfering RNA, morpholino) studies in various tissues, particularly the spinal cord [1,2]. More recently, similar protocols have been presented for use with *Xenopus *[3] and zebrafish [4-7], and somewhat more arduous technical methods can be used for *in utero *electroporation of mice [8,9]. All electroporation techniques are based on the application of an electric field to a tissue in the presence of a macromolecule of interest. The field induces transient pores in the plasma membrane of cells, as well as bulk flow of charged molecules toward one of the electrodes (for example, toward the cathode for negatively charged nucleic acids). This directional aspect of electroporation has been taken advantage of to unilaterally transfect the neural tube of chick, *Xenopus*, and zebrafish.

We began experimenting with electroporation of zebrafish in order to examine the development of commissural axon projections in the brain. Unilateral electroporation is an ideal technique as it allows one to visualize in detail the midline and contralateral behavior of commissural axons. In the transparent zebrafish embryo, it is possible to take time lapse movies of growth cone migration in transfected cells. In our studies we attempted to use existing electroporation techniques, but found them insufficient for our purposes for two reasons. First, we were examining the axonal projections of a mutant in which homozygous embryos could not be distinguished from wild-type siblings at the time of electroporation (1 day post fertilization (dpf)), thus only 25% of successful electroporations would be of interest. This meant a large number of embryos had to be electroporated, with the highest possible rate of success. Second, the axons of interest to us originated from small clusters of cells in the lateral forebrain, which necessitated a method that would reproducibly lead to the transfection of a large number of cells.

## Results and discussion

**Figure 1:**
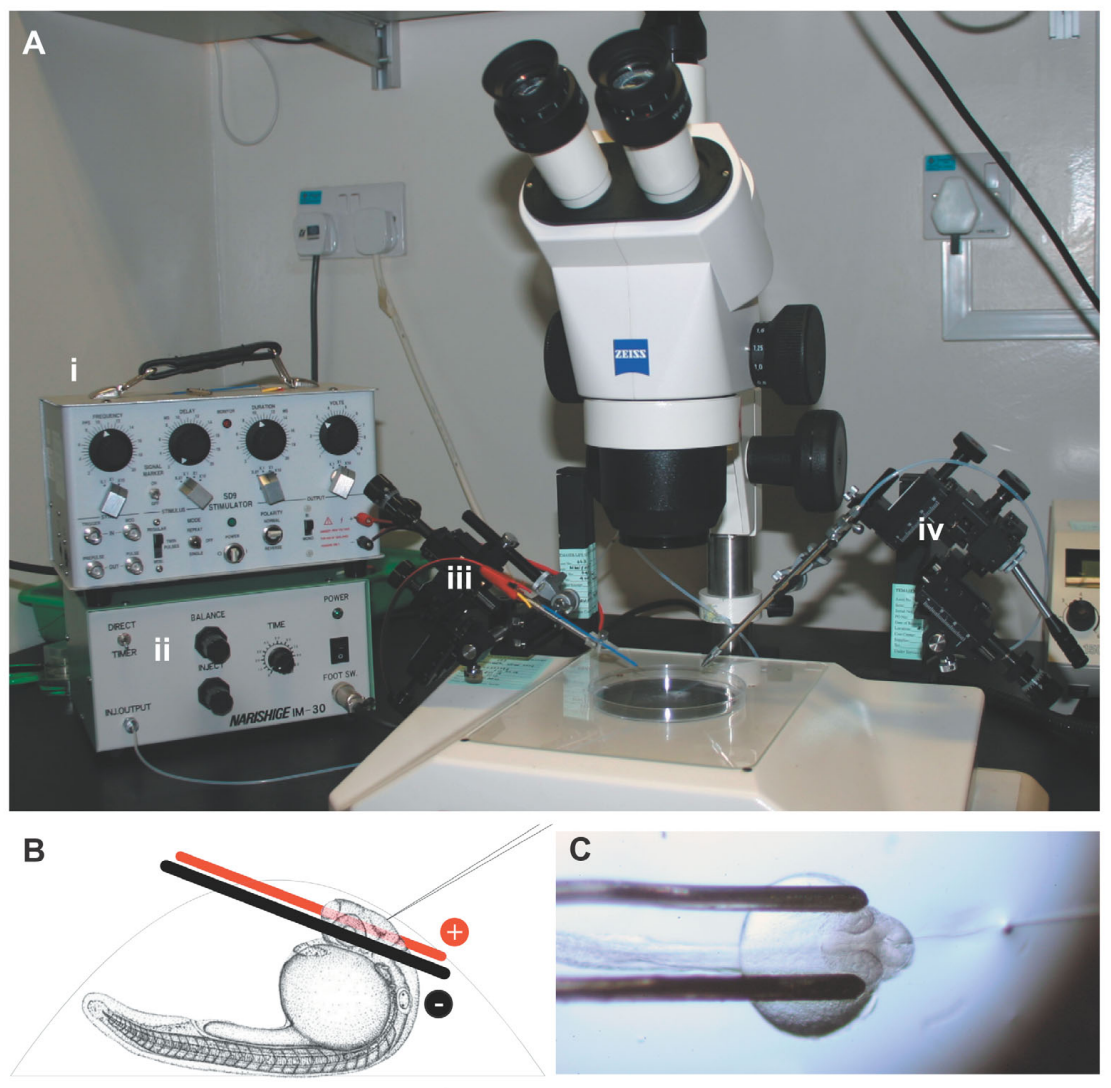
Electroporation apparatus. **(a) **The electroporation equipment assembled on a dissecting microscope: the Grass SD9 stimulator (i) and air pressure injector (ii) are connected to two micromanipulators controlling the electrodes (iii) and microinjection needle (iv). **(b) **Side view schematic of a 1 dpf zebrafish mounted in an agarose drop with the electrodes and injection needle in position. Electrodes are not drawn to scale. **(c) **Top view of an embryo mounted for electroporation, with electrodes in position and microinjection needle inserted into the brain ventricle.

After experimenting with variations on existing protocols for zebrafish brain electroporation [4,5], we obtained the most consistent results with the experimental set-up shown in Figure 1. We found that the electrode design parameters, mounting method, voltage, and the use of the GAL4/UAS system (a bipartite expression system based on the yeast GAL4 transcription factor, which drives expression of transgenes regulated by upstream activating sequences, UAS) were all critical to obtaining reproducibly high levels of expression in terms of number of cells, transgene levels within cells, and duration of expression. Pulse generation parameters did not seem to be critical to successful electroporation: single pulses and trains of pulses at various frequencies and durations gave similar results.

The equipment used (Figure 1a) is found in most developmental biology laboratories. The Grass SD9 stimulator is a basic, inexpensive square wave pulse generator that is simple to use. The electrodes are platinum iridium parallel bipolar electrodes built to custom specifications (see Materials and methods). Embryos electroporated at 20–24 hpf gave more consistent results than older embryos (not shown). Embryos were mounted yolk-up such that the brain area of interest was accessible to both the electrodes and microinjection needle (Figure 1b). One or both of the electrodes may be in contact with the embryo's eye(s). It is critical that embryos be mounted in individual agarose drops rather than multiple embryos mounted together in a larger volume of agarose (Figure 1c). With some practice, it is possible to electroporate up to 100 embryos in 1 hour with no lethality. When embryos did not survive the procedure, it was normally due to excessive damage with the microinjection needle or during removal from the agarose.

We compared the use of single plasmids with a two plasmid GAL4/UAS system, consisting of the neuronal HuC promoter driving GAL4 and enhanced green fluorescent protein (EGFP) or a transgene of interest driven by tandem UAS elements upstream of a basal fish promoter [10]. Both expression level and number of cells expressing transgenes at detectable levels were increased several-fold when the two-component system was used, compared to EGFP driven directly by cytomegalovirus (CMV), HuC, or α-tubulin promoters (not shown).

**Figure 2:**
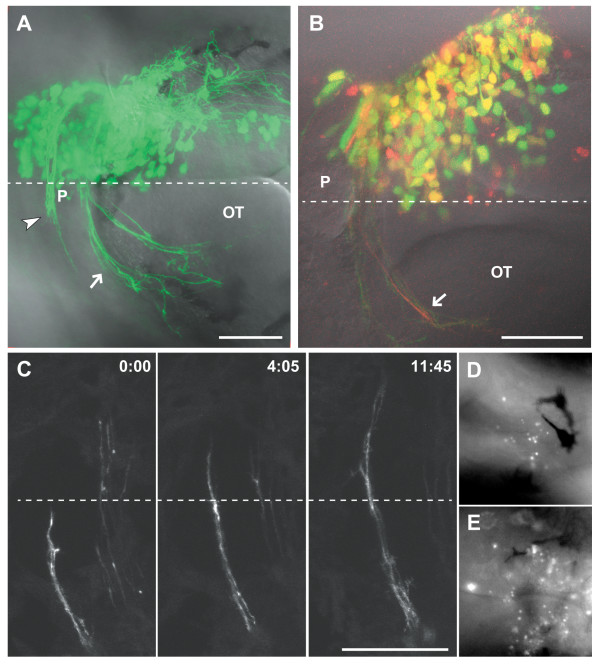
Results of electroporation at 2 dpf. **(a) **A 2 dpf embryo after electroporation at 24 hpf with 0.7 mg/ml each pHuC:GAL4/pUAS:EGFP. Axons of the developing habenular (arrowhead) and posterior (arrow) commissures are visible. **(b) **A 2 dpf embryo electroporated with 0.5 mg/ml each pHuC:GAL4/pUAS:EGFP/pUAS:mCherry. The mCherry channel (red) is less well resolved compared to EGFP (green) due to suboptimal excitation with a 543 nm laser line. Approximately 60% of cells express both transgenes at high levels (yellow). **(c) **Time series of commissural axons in the habenular commissure. Images were collected at room temperature, and growth cone migration is slower than normal. **(d,e) **Acridine orange staining two hours after electroporation shows higher levels of scattered cell death bilaterally in the brains of electroporated embryos (e) compared to unelectroporated siblings (d). Dorsal views, anterior to the left. Dashed lines indicate the midline. Time is hours:minutes. Scale bars = 50 μm. OT, optic tecum; P, pineal organ.

Electroporation of the right forebrain with pHuC:GAL4/pUAS:EGFP led to robust expression at 2 dpf and labeling of many contralaterally projecting axons (Figure 2a). Expression is visible under epifluorescence in most embryos as early as 3.5–4 hours after electroporation. If the injection site and electrode position are kept constant throughout an experiment, all embryos will show similar expression patterns. Table 1 shows results for a typical experiment. The voltage is important for high, reproducible expression levels with minimal lethality, with sharp decreases in transfection rates below 30 V. Lower voltages may be useful when fewer transfected cells are desired. Using volume rendering software, confocal z-stacks can be assembled into high-resolution reconstructions of the transfected neurons and their projections (Additional file 1). To test if two transgenes can be coexpressed in the same cells, we coelectroporated three plasmids: pHuC:GAL4/pUAS:EGFP/pUAS:mCherry (Figure 2b). While the majority of transfected cells expressed both fluorescent proteins, there is a wide range of relative expression levels, as well as cells that express detectable levels of just one transgene. By sampling from two coelectroporated embryos we estimated that roughly 60% of cells express both transgenes at high levels (see Materials and methods for details).

**Table 1:** Electroporation results at 48 hpf

	Transfection levels*				
		
Voltage	+++	++	+	None	Dead
30 (n = 77^†^)	72 (94%)	3 (4%)	-	-	2 (3%)
20 (n = 16)	2	8	5	1	-
10 (n = 16)	-	-	5	11	-

Injection of 0.7 mg/ml each pHuc:GAL4/pUAS:EGFP into the lateral forebrain and ventricle was followed by two manually applied trains of five 1 ms pulses at the indicated voltage. Transfection levels were greatly decreased with voltages lower than 30 V. *Embryos were viewed dorsally under a compound fluorescence microscope and categorized according to extent of transgene expression: +++, similar to Figure 2a or higher; ++, intermediate levels; +, few enough cells to make out and count individually, fewer than 20. ^†^Of 80 embryos electroporated in 2 experiments, 3 were killed by mechanical damage during removal from agarose and are not included.

We noted elevated levels of cell death, ascertained by staining with acridine orange, in electroporated embryos (Figure 2e). These cells were scattered bilaterally, suggesting that death was not a result of transfection but likely an effect of electric field application. Brain morphology and axon projections appeared normal in older electroporated embryos (Figures 2a–c and 4), suggesting that the increased cell death did not cause gross defects in brain development.

**Figure 3:**
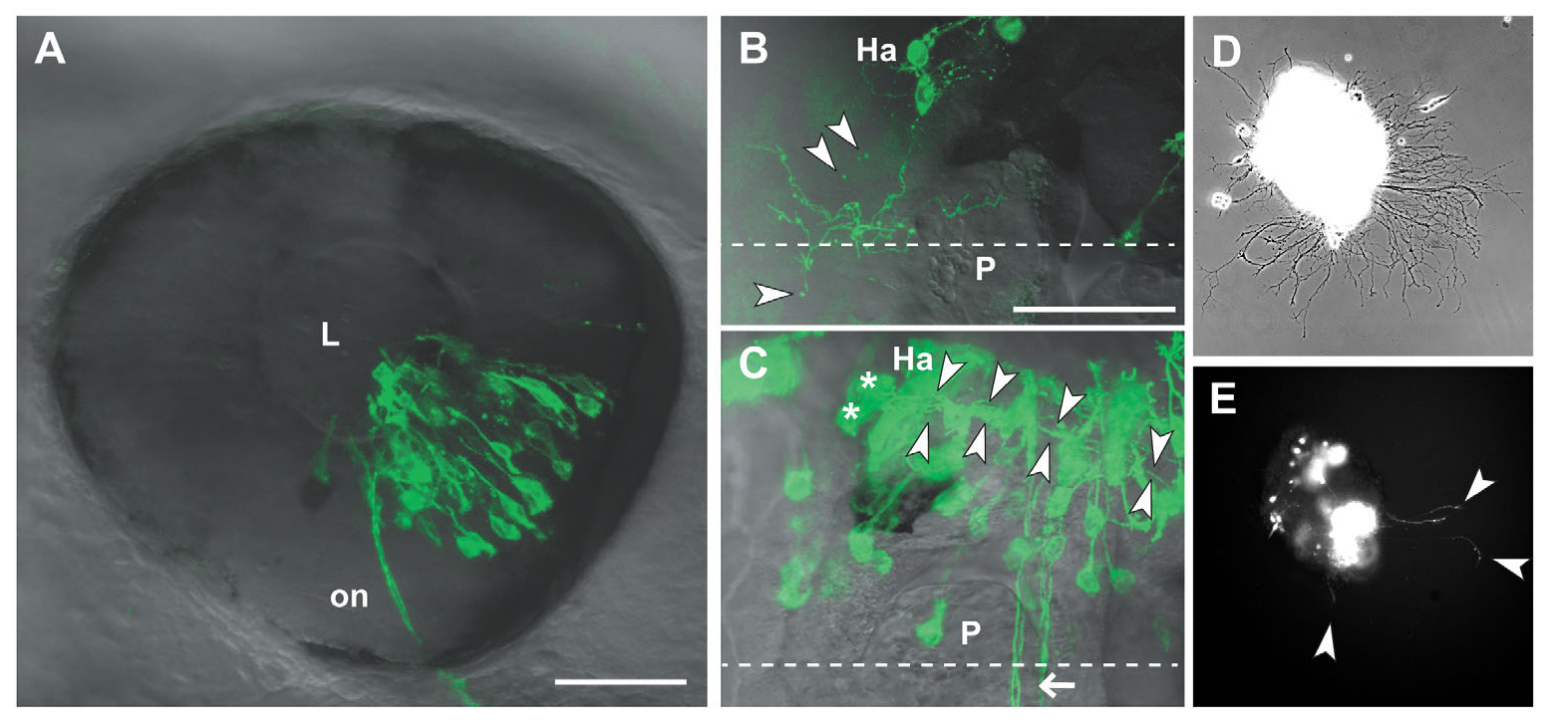
Analysis of transfected neurons *in vivo *and *in vitro*. **(a) **The eye of a 2 dpf embryo electroporated with pHuC:GAL4/pUAS:dnRyk-EGFP. Cells in multiple retinal layers are transfected in a distinct segment according to electrode positioning and injection site. Retinal ganglion cell axons are visible in the optic nerve (on). **(b) **A habenular (Ha) neuron contransfected with pHuC:GAL4/pUAS:dnEphB3-EGFP shows ectopic processes branching over the medial epithalamus, including the pineal organ (P). Extracellular exosome-like vesicles (arrowheads) are visible around the soma and processes. **(c) **Two habenular neurons (asterisks) expressing EGFP show the normal ventro-posterior projection into the fasciculus retroflexus (arrowheads). Commissural axons (arrow) are not derived from the habenula. **(d) **Bright field phase contrast and **(e) **fluorescence images of a 2 dpf forebrain explant from an embryo electroporated with pHuC:GAL4/pUAS:EGFP after 12 hours in culture. EGFP positive neurons and axons (arrowheads) can be tracked over time. Anterior is to the left in (a) (lateral), (b,c) (dorsal). Dashed lines indicate the midline. Scale bars = 50 μm. L, lens.

**Figure 4:**
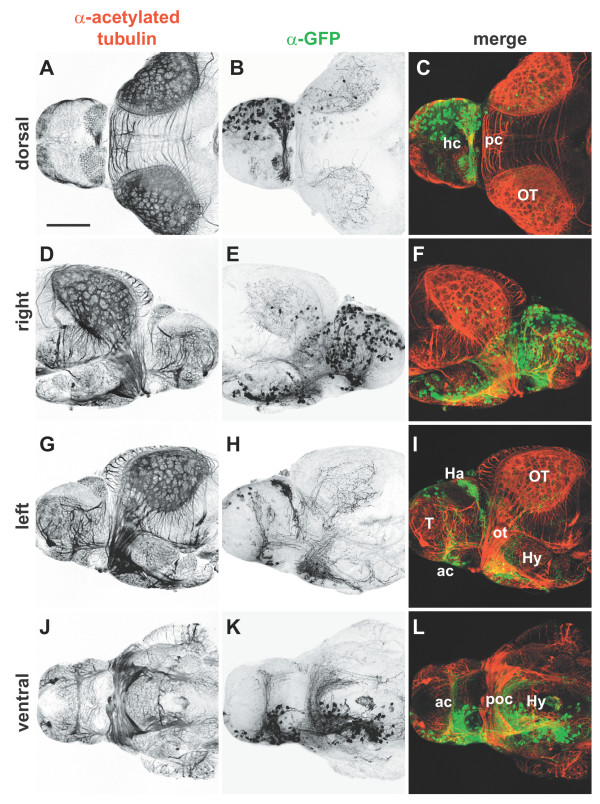
Whole mount immunocytochemistry of electroporated brains. Isolated 4 dpf brains from embryos in which pHuC:GAL4/pUAS:EGFP were unilaterally electroporated into the right forebrain. Brains were stained with **(a,d,g,j) **anti-acetylated tubulin to label axons (red) and **(b,e,h,k) **anti-GFP to mark transfected cells (green); **(c,f,i,l) **merged images. Widespread expression of EGFP is seen in neuronal cell bodies of the right telencephalon and diencephalon (b,e). All major commissures contain labeled fibers and contralateral axonal projections can be seen in detail in left and ventral views (h,k). Anterior is to right in (d-f), to the left in all other panels. Scale bar = 100 μm. Ac, anterior commissure; Ha, habenula; hc, habenular commissure; Hy, hypothalamus; ot, optic tract; OT, optic tectum; pc, posterior commissure; poc, postoptic commissure; T, telencephalon.

Zebrafish embryos are transparent, allowing for *in vivo *time lapse imaging of fluorescently labeled cells and structures. This has been taken advantage of to capture the dynamics of commissural axons using lipophilic tracer dyes [11] and transgenic lines [12]. Electroporation allows the extension of these techniques to the analysis of the effects of specific transgenes on growth cone dynamics. We used electroporation of pHuC:GAL4/pUAS:EGFP to make time lapse movies of the zebrafish habenular commissure, which forms in the dorsal diencephalon at around 45–48 hpf (Figure 2c; Additional file 2). Expression of various transgenes will allow us to test the requirements of specific signaling pathways for midline crossing.

Our experimental objective was to test transgenes predicted to affect axon guidance. To this end, we made a Gateway expression vector to facilitate the rapid cloning and expression of genes of interest as carboxy-terminal EGFP fusion proteins under the control of UAS. We have tested dominant negative receptor constructs, where the cytoplasmic domain of a receptor of interest has been replaced with EGFP. We constructed receptor fusions for canonical axon guidance receptor classes including Ephs, Deleted in Colorectal Carcinoma (DCC), and Robos. Ryk, a vertebrate homolog of *Drosophila *Derailed, is a Wnt receptor with several functions in axon guidance, including the topographic mapping of retinal axons [13,14]. We constructed a dominant negative zebrafish Ryk receptor fused to EGFP (dnRyk:EGFP) and used electroporation to express it in a subset of retinal ganglion cells (Figure 3a). The resulting tectal projections (not shown) were easily observed *in vivo*.

The Eph receptor tyrosine kinases constitute a large class of axon guidance receptors. We made dominant negative Eph receptors, including EphB3. Expression of dnEphB3-EGFP in a habenular neuron affected axonal behavior. Habenular neurons normally project ventroposteriorly via the fasciculus retroflexus to the interpeduncular nucleus [15-17]. Figure 3b shows a habenular neuron expressing dnEphB3-EGFP, which caused abnormal process branching and elaboration over the epithalamus, including into the pineal organ. In other cases, we observed axons of normal morphology within the habenular commissure; however, axons of this commissure derive from the lateral diencephalon and not the habenulae [18]. Habenular neurons expressing EGFP project normally (Figure 3c). In cases of dnEphB3-EGFP expression, we observed small, fluorescently labeled vesicles that appeared to be derived from axons. We did not observe these vesicles with EGFP or with dnRyk-EGFP, but did see similar structure when axons were labeled with lipophilic membrane dyes such as DiI (not shown). Interestingly, EphB-containing vesicles derived from cultured hippocampal neurons have recently been reported [19]. Electroporation followed by *in vivo *imaging thus provides evidence that such vesicles are produced in the embryo and may be derived from specific domains of the axonal membrane.

Primary neuronal culture has been used successfully to explore axon guidance mechanisms. Growth cone migration *in vitro *can be assayed in the presence of exogenously provided cues. We have tried lipofection-mediated transfection of zebrafish primary neuronal cultures without success (data not shown). The high transfection rate of electroporation, in this case performed bilaterally, allowed the easy identification of many transgene-expressing neurons in cultured brain explants (Figure 3d,e).

While the dorsal regions of the brain are easily viewed *in vivo*, ventral and some lateral structures are obscured by the eyes, jaw, and gills. Removing the brain followed by fixation and antibody staining allows for high resolution imaging of the entire brain, but is somewhat tedious for large numbers of embryos. Testing various fixation and permeabilization protocols for immunocytochemistry, we serendipitously found that large numbers of brains can be isolated easily from embryos by fixation in trichloracetic acid (TCA) followed by collagenase digestion. These brains are suitable for whole mount antibody staining, though not all antigens are well preserved after TCA fixation and tissue morphology is indistinct under bright field optics. Labeling fixed whole mount brains with anti-GFP and anti-acetylated tubulin antibodies allows for comparison of the axonal projections of transfected neurons relative to the overall neuroanatomy of the brain (Figure 4). A lateral view of the right side shows the transfected cell bodies, primarily in the telencephalon and diencephalon (Figure 4e). From the left, the resulting contralateral projections can be analyzed (Figure 4h).

Some technical problems remain, which we are addressing. Electroporation of embryos older than 24 hpf produced inconsistent results and overall lower numbers of transfected cells. However, some transgenes we expressed appeared to disrupt neuronal differentiation or cell survival and we would like to express them later. The use of heat shock promoters driving GAL4, either on a separate plasmid or in a stable transgenic background, so that expression can be temporally controlled is a promising solution.

Another issue is that some transgenes we tested appeared to be regulated post-transcriptionally and spatially, and were only found in particular subcellular compartments, within distinct puncta, or were present at low levels. In these cases, the entire morphology of a transfected neuron was difficult to image, and time lapse imaging was impractical. Ideally, we would like to coexpress a red fluorescent protein to label the entire neuron. Because coelectroporating two UAS vectors with the GAL4 driver led to inconsistent coexpression, we are testing Gateway expression vectors that contain two independent UAS elements: one driving the cytosolic red fluorescent protein while the other drives the EGFP fusion construct of interest.

## Conclusion

The electroporation method presented here allows for the simple and efficient introduction of transgenes of interest into populations of neurons. Existing methods of electroporation of zebrafish neurons are effective, but have significant rates of lethality and unsuccessful transgenesis. Cerda and colleagues [4] demonstrated the electroporation of mRNA and morpholinos in addition to DNA, and were able to transfect several different tissues, including brain, retina, somites, and trunk. In our hands, the independent manipulation of the electrodes and the insertion of one electrode into the embryo led to frequent lethality and difficulty in reproducibly transfecting the same cells in multiple embryos. On the other hand, the independent positioning of electrodes of this technique allows for better targeting of the electric field compared to our relatively large external electrodes. The protocol presented here has the advantage of simple electrode placement and of allowing a larger number of embryos to be transfected in a uniform manner. As it relies on commercially available electrodes and basic equipment, it should be easily reproducible in any lab. We anticipate that it will be possible, with slight modifications, to transfect different tissues and to deliver charged morpholinos. It may also be possible to restrict expression to more precise areas by controlling the volume and position of plasmid injection.

We found the critical parameters for success to be the choice of electrodes, the embryo mounting method, and use of the GAL4/UAS expression system. In these experiments, separate plasmids were used. There is increasing availability of stable transgenic zebrafish lines expressing GAL4 under various promoters. Use of these lines will allow for single UAS plasmid electroporation, which should increase efficiency.

In addition to *in vivo *still and time lapse imaging, we present two ways the electroporation method can be used with further analytical tools. Primary neuronal culture is a powerful system for studying the cell biology of axon growth and guidance. Electroporation allows these *in vitro *techniques to be coupled with transgenesis. Transfected and non-transfected axons from the same explant can be compared in their responses to exogenously applied factors. The enzymatic brain isolation technique is a useful way to collect large numbers of samples. In this case, we have used it to image in detail the results of a routine electroporation. It is also useful for the analysis of mutant phenotypes, screening, or other situations where the number of brains needed makes manual dissection impractical. The zebrafish is a well-established model for vertebrate developmental biology. The methods presented here will extend its utility for studies of the development of the embryonic nervous system.

## Materials and methods

### Equipment

A Zeiss (Oberkochen, Germany) dissecting scope was fitted on opposite sides with two Narishige (Tokyo, Japan) micromanipulators, one to manipulate the electrodes (left) and the other the injection needle (right) (Figure 1). A Narishige air pressure injector was used for DNA delivery. A Grass (West Warwick, Rhode Island, USA). Telefactor SD9 voltage stimulator was connected to the electrodes to provide pulses. Custom platinum iridium parallel bipolar electrodes 125 μm in diameter and spaced 500 μm apart were used for electroporation (catalog #PB-SA0575, FHC, Bowdoinham, Maine, USA). During electroporation, the electrodes can develop deposits of dried agarose. Between or during long experiments the electrodes were cleaned with methanol and a soft brush, or using a commercially available ultrasonic jewellery cleaner.

### Zebrafish

Zebrafish were maintained according to standard procedures and in accordance with Institutional Animal Care and Use Committee guidelines. Electroporation was done at 18–24 hpf. A significant reduction in efficiency of transfection was observed in older embryos. Some embryos were raised in E3 containing 0.003% 1-phenyl-2-thiourea to prevent melanin synthesis.

After being dechorionated with forceps, embryos were anesthetized in electroporation Ringer's (180 mM NaCl, 5 mM KCl, 1.8 mM CaCl_2_, 5 mM Hepes pH 7.2), as described by Cerda *et al*. [4], containing 0.016% 3-amino benzoic acid ethyl ester (MS222; Sigma-Aldrich, St. Louis, Missouri, USA). Groups of 6–10 embryos were immersed briefly in 37°C 1% low melting point agarose (LMPA; Bio-Rad, Hercules, California, USA) in electroporation Ringer's. The embryos were removed from the agarose with a glass pipette and placed on an inverted plastic Petri dish lid in individual drops of LMPA. As the agarose cooled, they were maneuvered into the desired orientation using a tungsten needle. It is essential that the embryos be in individual drops, the drops should be as small as possible, and the orientation consistent to facilitate injections and electrode positioning.

Each group was immediately electroporated after orientation so that the agarose was still somewhat soft, which eased the insertion of the electrodes. After electroporation, the embryos were covered with E3 embryo medium. At the end of the experiment, all embryos were covered with E3 for approximately 15–30 minutes to recover from anesthetic. The LMPA was peeled away from the head and yolk with a tungsten needle. The embryos often wriggle free at this stage, or can be removed by gentle aspiration with a glass pipette.

### Plasmid DNA

The pHuC:GAL4-VP16 plasmid used to drive neuronal expression has been described [20]. pUAS:EGFP was made by replacing the CMV promoter from pEGFP-N1 (Invitrogen, Carlsbad, California, USA) with a promoter consisting of 14 UAS elements upstream of a basal fish promoter [10], and pUAS:mCherry by subsequent replacement of EGFP in this plasmid with mCherry from pRSETB-mCherry [21]. Gateway cloning products were from Invitrogen. The pUAS:rfC.1-EGFP Gateway expression vector was made for expressing genes of interest fused to the amino terminus of EGFP. The RfC.1 cassette from the Gateway Vector Conversion kit was ligated into the *Sma*I site of pUAS:EGFP. Entry clones were made using pENTR/D-TOPO and pCR8/GW/TOPO cloning kits using *Pfu *(Stratagene, San Diego, California, USA or *Taq *(Expand High Fidelity, Roche, Basel, Switzerland) polymerase PCR products, respectively. Gateway recombination reactions were done with LR clonase according to the manufacturer's instructions but using one-quarter of the recommended reaction components and volume. Because pENTR/D-TOPO and pUAS:RfC.1-EGFP are both kanamycin resistant, the entry clone was linearized at a unique restriction site either prior to or after recombination. For electroporation, roughly equimolar amounts of circular GAL4 and UAS plasmid DNA were used to a total concentration of 1–2 mg/ml.

pUAS:dnEphB3-EGFP was made by amplifying a segment of the zebrafish *ephb3 *coding sequencing (GenBank:NM_131097) corresponding to the extracellular and transmembrane domains by RT-PCR from 3 dpf zebrafish RNA (forward primer, 5'-CACCATGGATTATTCGCTGTTATTATAC-3'; reverse primer, 5'-CGGATCCTCGTAGGTGAAAG-3'). The PCR product was cloned into a Gateway entry vector and confirmed by sequencing. The entry clone was used for LR recombination into pUAS:RfC.1-EGFP (see above). pUAS:dnRyk-EGFP (GenBank: XM_678748) was made in the same way (forward primer, 5'-CACCATGTTTCTGCCAGCGCGG-3'; reverse primer, 5'-AAACACGTAGGCCCCCAAAGC-3').

DNA constructs were prepared using Qiagen (Hilden, Germany) mini or midi prep kits, followed by concentration by either by sodium acetate/ethanol precipitation or evaporation in a heated vacuum centrifuge to the desired concentration in 10 mM Tris. These constructs and their sequences are available upon request.

### DNA injection and electroporation

Glass injection needles were pulled from capillaries (1.0 mm OD, 0.78 mm ID, with filament) on a Flaming-Brown P97 puller (Sutter Instruments, Novato, CA, USA) and back loaded with DNA solution. The needle tip was broken off with forceps, and oriented as in Figure 1. The electrodes were positioned first using the left manipulator, followed by insertion of the microinjection needle with the right manipulator. DNA was injected into the area of interest using a Narishige M30 air pressure injector. Enough DNA was injected such that swelling of the brain ventricle was observed. Immediately, five 30 V pulses, each lasting 1 millisecond were applied manually using a Grass SD9 stimulator, leading to electroporation of the side of the brain near the cathode. Injection/pulsing was often repeated for other injection sites or to increase transfection rate. Likewise for bilateral electroporation, the polarity of the stimulator was reversed and injection/pulsing was repeated. Electrode position was the same for retinal electroporation, but DNA was injected directly into the retina.

### Brain explant culture

At 2 dpf, bilaterally electroporated embryos were anaesthetized in Ringer's solution containing MS222 and 1 mg/ml bovine serum albumin (BSA). Their brains were removed using electrolytically sharpened tungsten needles and cut into small clumps. A fire-polished microinjection needle with a wide bore opening was used to transfer brain clumps to a small drop of culture medium with a mouth pipette (as a washing step). The clumps were then transferred to a poly-lysine-coated cover slip bottom dish (Matek, Ashland, Massachussetts, USA) containing 2 ml of culture medium. Media reagents were from Sigma: L15 containing 1% N1 neuronal growth supplement, 10 mM Hepes, and 1% GPS (glutamine/penicillin/streptomycin). Explants were cultured for 8 hours or overnight at 28°C.

### Brain isolation

Embryos at 3 dpf or older were fixed in 2% TCA in PBST (phosphate-buffered saline + 1% Triton X-100) for several hours at room temperature or overnight at 4°C. After several washes in PBST, the embryos were treated with 1 mg/ml collagenase (Sigma) in PBS for 10–20 minutes on a nutator. After 20 minutes or if they started to fall apart, embryos were washed several times with PBST to stop collagenase digestion. They were then transferred to a Petri dish and gently swirled in PBST. During this process, the embryos would fall apart, leaving individual eyes and brains intact. Brains often remained attached to the spinal cord.

### Immunocytochemistry and staining

Isolated brains were washed in blocking solution (5% normal goat serum/2% BSA in PBST) for one hour. Primary antibody incubation was done in blocking solution at 4°C overnight on a nutator, followed by several washes in PBST. Secondary labeling was done overnight at 4°C or several hours at room temperature. Anti-acetylated tubulin (Sigma) and anti-GFP (Torrey Pines, Houston, Texas, USA) were used at 1:1,000. Alexa488 anti-rabbit and Alexa568 anti-mouse secondary antibodies (Invitrogen) were used at 1:500. To detect cell death, embryos were incubated with 5 μg/ml acridine orange (Sigma) for 30 minutes, followed by washing in E3 and examination under epifluorescence.

### Imaging and analysis

Live embryos and whole mount brains were imaged on a Zeiss LSM510 confocal microscope using a 40× 0.8 NA water immersion objective. Cultured explants and acridine orange stained embryos were imaged on a Zeiss Axiovert 200 M using a 40× phase contrast objective, a SPOT Insight CCD camera (Diagnostic Instruments, Sterling Heights, Michigan, USA) and MetaMorph software (Molecular Devices, Sunnyvale, California, USA). Projection of confocal z-stacks was done using Zeiss software and NIH ImageJ[22]. Volume rendering was done with Volocity (Improvision, Coventry, England). Photographs of mounted fish were taken with a Canon EOS E400. Live fish were anesthetized and mounted in 1% LMPA/E3. For brains, mounting chambers were made on a glass slide by affixing layers of electrical tape and cutting out a small square. A brain in PBS was dropped into the chamber and a cover slip placed over it. The depth of the chamber was determined such that moving the cover slip rotated the brain to the desired orientation.

Cotransformation rates (Figure 2b) were estimated using NIH ImageJ. Confocal stacks were separated by channel and independent thresholds applied to allow for easy counting of high intensity cells (cells with contiguous label above threshold at a level that eliminates all background). Colocalization of pixels above threshold was scored as a cotransfected cell. Four randomly chosen non-overlapping planes from confocal stacks of two embryos were analyzed (142 cells total).

## Competing interests

The author(s) declare that they have no competing interests.

## Authors' contributions

MH performed the experiments. MH and SJ conceived of and designed the experiments, interpreted the results, and wrote and approved the manuscript.

## Supplementary Material

Additional file 1 Three-dimensional volume rendering of electroporated neurons. A volume rendering of a confocal stack of a live 3 dpf embryo electroporated with pHuC:GAL4/pUAS:EGFP Click here for file

Additional file 2 Time lapse of commissural axons. Time lapse series (7.5 minutes/frame) of confocal z-projections of habenular commissural axons crossing in a 2.5 dpf embryo unilaterally electroporated with pHuC:GAL4/pUAS:EGFP Click here for file
